# AIS-Based Radar Error Correction Using a Vision Transformer Variant for Range and Azimuth Error Reduction

**DOI:** 10.3390/s26123782

**Published:** 2026-06-13

**Authors:** Zhaohui Fan, Gandong Liu, Bo Peng, Jinyong Chen

**Affiliations:** 1 China Electronics Technology Group Corporation 54th Research Institute, Shijiazhuang 050081, China; 202420000705@std.uestc.edu.cn (Z.F.); pengbocti54@163.com (B.P.); 2 Glasgow College, University of Electronic Science and Technology of China, Chengdu 611731, China; 2022190907014@std.uestc.edu.cn

**Keywords:** radar error correction, systematic error estimation, AIS data, Vision Transformer variant

## Abstract

Shore-based maritime surveillance radars suffer from systematic range and azimuth errors that degrade target-tracking accuracy. This paper proposes a Vision Transformer (ViT) variant that corrects these errors using Automatic Identification System (AIS) data as the ground truth, modelling nonlinear error patterns via self-attention without requiring explicit physical models of the underlying error sources. Evaluated on the Maritime Target Detection and Tracking (MTDSP) dataset (≈80,000 paired radar-AIS observations), the proposed method reduces range mean absolute error (MAE) by 98.5% (514.76 m → 7.77 m) and azimuth MAE by 89.8% (1.37° → 0.14°) relative to uncalibrated measurements. Controlled experiments isolating architectural components confirm that self-attention, patch embedding, and multi-task learning each contribute measurable gains, particularly in tail-error robustness. These results demonstrate the viability of Transformer-based architectures for correcting radar systematic errors in maritime surveillance.

## 1. Introduction

Maritime surveillance systems are critical for ensuring navigational safety, maritime security, and environmental protection [1]. Among the sensing technologies used in these systems, shore-based radar remains a primary tool for tracking moving maritime targets because of its wide coverage and all-weather capability. However, radar measurements are inherently subject to systematic errors [2], particularly in the range and azimuth dimensions, which can significantly degrade target-tracking accuracy and lead to erroneous situational awareness [3]. Radar error registration—the identification and correction of these systematic biases—is therefore essential to improving the reliability of maritime surveillance systems.

Physical origins of systematic radar errors. The systematic errors addressed in this work arise from several physical mechanisms. Range errors are primarily caused by atmospheric refraction—where the radar beam bends as it passes through air layers of varying density, producing a discrepancy between the measured line-of-sight distance and the true geometric range—and by timing offsets in the radar’s signal processing chain. Azimuth errors originate predominantly from antenna pointing inaccuracies, including mechanical misalignment of the radar pedestal relative to true north, gear backlash in the rotation mechanism, and wind-induced antenna deformation. Both error dimensions are further modulated by environmental factors. Temperature and humidity gradients alter the atmospheric refractive index profile. Sea surface reflections produce multipath propagation, where the direct-path echo and the sea-reflected echo interfere at the receiver, shifting the apparent target position. Evaporation ducts—a common phenomenon in maritime environments where a strong humidity gradient traps radar energy in a surface waveguide—can extend detection range but introduce height-dependent ranging biases. Sea clutter (wind-driven wave returns) introduces spurious detections that can bias the centroid of the target echo. Vessel motion (roll, pitch, yaw) further changes the effective radar cross-section and the apparent centroid of the reflected signal. Critically, these errors are not random—they exhibit systematic, spatially coherent patterns that are deterministic functions of the radar measurement geometry (range *r* and azimuth θ) and the prevailing environmental state. This deterministic structure is what makes data-driven error correction possible: a model that learns the mapping from (r,θ) to the expected systematic error can predict and compensate for these biases without requiring explicit physical models of each contributing mechanism.

Terminological note:In radar engineering, “calibration” typically refers to hardware-level adjustments (e.g., antenna alignment, timing circuit tuning, receiver gain setting). The present work addresses a distinct but related problem: software-based systematic error correction, where a learned model predicts and compensates for measurement biases using AIS data as a positional reference, without modifying any physical radar component. Throughout this paper, we use the terms “error correction” and “error registration” to describe our approach and reserve “calibration” for references to hardware calibration in the existing literature. This distinction is important because the two problems differ fundamentally in their assumptions, methods, and deployment constraints.

The primary sources of radar errors include sensor calibration imperfections [4], environmental factors such as atmospheric refraction and sea clutter, antenna pointing errors, and timing delays in signal processing. These errors typically manifest as consistent biases rather than random noise, making them amenable to correction using external reference sources. The Automatic Identification System (AIS), a mandatory vessel transponder system operating on Very High Frequency (VHF) bands, provides high-precision vessel position, velocity, and heading information with a typical positioning accuracy of 5–10 m [5,6]. Because AIS measurements are independent of radar sensing mechanisms and generally offer superior spatial accuracy, AIS has emerged as an ideal reference source for radar error correction [7]. By using AIS data as the ground truth, researchers can effectively estimate and correct radar errors, improving target localisation precision for maritime safety and situational awareness [8].

A substantial body of research has been devoted to radar systematic error estimation and correction, including methods that use AIS data. These methods can be broadly grouped into four categories: spatial partitioning methods, multi-station systems, data-fusion techniques, and optimisation-based approaches. Although each category has contributed important advances, they share inherent limitations that motivate the development of more powerful error correction frameworks.

Spatial partitioning methods divide the surveillance area into regions according to error characteristics to improve correction accuracy. For instance, Dong et al. [9] proposed a non-uniform partitioning method that adapts region boundaries to the local error distribution, improving correction accuracy in spatially heterogeneous conditions. However, such methods rely heavily on manual or heuristic region design, making them sensitive to the choice of partitioning granularity. When the underlying error distribution is spatially complex or varies over time, fixed partitioning schemes cannot adapt accordingly, leading to suboptimal correction in dynamic maritime environments.

Multi-station systems leverage multiple radar stations to improve error-correction performance through cooperative measurements. Ma et al. [10] proposed the Multi-Station Azimuth-based Position Error Calibration Algorithm (MAPEC) for distributed multi-station systems. Their method addresses mixed amplitude-phase errors in direct positioning by converting estimated errors into positions on the localisation grid and adjusting steering vectors, achieving significantly improved localisation accuracy under complex error conditions. Nevertheless, multi-station approaches require deploying and coordinating multiple radar stations, which imposes substantial infrastructure costs and limits their applicability to scenarios where only a single radar station is available.

Data fusion techniques integrate information from multiple sources to estimate and correct systematic errors in radar data [11]. Sansot et al. [12] proposed a radar auto-calibration approach based on Kalman-filter data fusion that combines complex gain estimates from multiple calibration cycles, reducing reliance on costly hardware while maintaining sustained error-correction accuracy. Jiang et al. [13,14] introduced a multi-target joint error estimation method based on real-time AIS data, demonstrating the benefits of leveraging multiple targets for calibration. Tao et al. [15] proposed a radar error calibration method based on ADS-B data that jointly corrects radar and ADS-B discrepancies, including both fixed ADS-B position errors and target echo centre variations, thereby improving the precision and stability of systematic error estimation. Liu et al. [16] developed a spatial error registration algorithm based on probability hypothesis density filtering to address the challenge of data association in multi-target scenarios. Despite their effectiveness in combining heterogeneous data sources, these fusion-based methods typically employ linear or weakly nonlinear models (e.g., Kalman filters) for error estimation, which limits their ability to capture the complex, spatially varying nonlinear error patterns commonly observed in real-world radar systems.

Optimisation-based approaches formulate radar calibration as an optimisation problem for estimating systematic errors. Li et al. [17] proposed a Specific Iterative Closest Point (SICP) algorithm for estimating 2D radar system errors using ADS-B data, eliminating the need for temporal registration and reducing dependence on sensor-data accuracy, while achieving faster and more accurate error estimation than conventional methods. Jia et al. [18] investigated sensor-error calibration with imperfect calibrators, proposing a weighted least-squares solution and analysing optimal calibrator-sensor geometries, thereby providing theoretical guidance for calibrator deployment in practical applications. Zhai et al. [19] proposed a radar automatic calibration method based on unmanned aerial vehicles (UAVs), addressing the high cost and unreliable truth-value acquisition associated with traditional methods while providing real-time calibration with improved generality, efficiency, and accuracy. Liu et al. [20] developed a radar calibration method based on cooperative targets with an integrated measurement-error model, using an improved sparrow search algorithm (ISSA) to estimate systematic errors without linearising the underlying nonlinear equations; this avoids the accuracy loss inherent in linearisation-based approaches. While optimisation-based methods can achieve high accuracy under well-defined conditions, they generally rely on simplified parametric error models and are susceptible to local optima. Their performance degrades when error patterns exhibit strong spatial heterogeneity or temporal variability, conditions that are common in dynamic maritime scenarios [21].

In summary, traditional methods share a common limitation: they rely on explicit, often simplified, error models that cannot fully capture the spatially varying, nonlinear error patterns observed in real-world radar systems. This gap motivates the exploration of data-driven approaches that learn error representations directly from observations.

Recent advances in deep learning have significantly influenced radar signal processing. Geng et al. [22] comprehensively reviewed applications such as radar waveform design, target recognition, and jamming suppression, highlighting both the strengths and limitations of deep learning in radar systems. Transformer-based architectures have shown particular promise: Lu et al. [23] proposed RD-Transformer for coherent detection under low signal-to-noise ratio (SNR) conditions, achieving 14–20 dB SNR improvement over traditional methods; Tang et al. [24] developed TFGM-RMNet for radar signal recognition, maintaining 97% accuracy at SNR = −10 dB.

However, applying deep learning specifically to radar systematic error correction remains largely unexplored. While general-purpose function approximators such as Deep Neural Networks (DNNs) [25] can learn nonlinear mappings from data, they process each input through fixed-size fully connected layers, which limits their ability to capture complex feature interactions and long-range dependencies among input variables. Furthermore, single-task DNNs treat range and azimuth error prediction as separate problems, failing to exploit the inherent correlation between these two error dimensions. To the best of our knowledge, no prior work has applied Transformer-based architectures to the problem of AIS-based radar systematic error correction.

The Vision Transformer (ViT) [26], originally proposed for image classification, offers a fundamentally different computational paradigm through its self-attention mechanism [27]. Unlike DNNs, which rely on local feature extraction via fixed weight matrices, the self-attention mechanism computes pairwise interactions across all positions in the input token sequence, enabling the model to capture global dependencies among input features regardless of their positions in the feature vector. This property is particularly well-suited for radar error correction, where the error at a given measurement point may depend on nonlinear interactions among range, azimuth, and derived trigonometric features. To bridge this gap, this paper introduces a radar error correction framework based on a ViT variant adapted from image classification to tabular regression, termed RadarCal-ViT. The proposed approach offers several distinct advantages over existing methods. First, the self-attention mechanism captures complex nonlinear dependencies among the engineered features that traditional parametric models and shallow learning methods cannot represent. Second, the patch embedding strategy automatically learns hierarchical error representations by grouping related features, without requiring manual spatial partitioning of the surveillance area. Third, the multi-task prediction heads simultaneously estimate range and azimuth errors, exploiting their inherent correlation to improve overall accuracy. Fourth, the lightweight architecture (≈205,000 parameters) requires only CPU-level computational resources for both training and inference, making it readily deployable on resource-constrained edge devices commonly used in maritime surveillance systems.

To validate the proposed method, the Maritime Target Detection and Tracking (MTDSP) dataset [28] is employed, providing multi-source observations of maritime targets, including synchronised radar and AIS data. This dataset enables comprehensive evaluation across diverse maritime scenarios, supporting a realistic assessment of the method’s robustness. Comparative experiments with traditional error-correction methods and alternative machine learning approaches (e.g., Single-Task Deep Neural Network (STDNN), Support Vector Regression (SVR) [29], and XGBoost [30]) demonstrate the ViT variant’s effectiveness in reducing both range and azimuth errors.

Scientific novelty. The primary contribution of this work is not the Transformer architecture itself—which builds on well-established ViT designs—but rather the demonstration that (i) radar systematic error correction can be formulated as a tabular regression problem amenable to self-attention; (ii) the 12 engineered features (Section 2.3.3) provide a physically motivated representation that captures the geometric and trigonometric structure of radar errors without requiring environmental sensor data; and (iii) when capacity and training are properly controlled, specific architectural choices (patch embedding of feature groups, CLS token aggregation, and uncertainty-weighted multi-task learning) yield measurable improvements in tail-error robustness (P95) that are statistically significant and practically meaningful for maritime safety. We do not claim that the ViT variant is universally superior to all alternatives; rather, we provide a controlled experimental framework that quantifies precisely which design choices matter and by how much.

The remainder of this paper is organised as follows: Section 2 describes the data preprocessing pipeline and the proposed ViT variant architecture. Section 3 presents the experimental setup and results, comparing the ViT variant with baseline methods. Section 4 discusses the implications of the results and potential extensions. Section 5 concludes the paper with the key findings and future research directions.

## 2. Materials and Methods

This section first formalises the radar error correction problem, then describes the dataset, the data preprocessing pipeline, the proposed ViT variant architecture, and the training procedure.

### 2.1. Problem Formulation

Let a shore-based radar station located at surveyed position $\left(\phi_{0},\lambda_{0},h_{0}\right)$ produce a sequence of measurements ${\left\{m_{i}\right\}}_{i=1}^{N}$, where each measurement $m_{i}=\left(r_{i},\theta_{i}\right)$ consists of a range $r_{i}\in \left[r_{\min},r_{\max}\right]$ and an azimuth angle $\theta_{i}\in \left[0^{\circ },360^{\circ }\right)$. Simultaneously, vessels in the surveillance area broadcast AIS messages containing their geographic positions $\left(\phi_{i},\lambda_{i}\right)$ at a coarser temporal resolution. After spatial alignment (Equations (4) and (5)) and temporal synchronisation (Equations (6) and (7)), each radar measurement is paired with an AIS-derived reference position $\left(r_{i}^{\mathrm{AIS}},\theta_{i}^{\mathrm{AIS}}\right)$. The systematic error at measurement *i* is:

(1) $$\Delta r_{i}=r_{i}-r_{i}^{\mathrm{AIS}},\;\Delta \theta_{i}=\theta_{i}-\theta_{i}^{\mathrm{AIS}}$$

The radar error correction problem is then formulated as follows: **given** a training set $D={\left\{\left(x_{i},\Delta r_{i},\Delta \theta_{i}\right)\right\}}_{i=1}^{N}$, where $x_{i}\in R^{d}$ is a feature vector derived from the raw radar measurement (Section 2.3.3), **find** a function $f_{\Theta }:R^{d}\to R^{2}$ parameterised by Θ that predicts both errors simultaneously:

(2) $$f_{\Theta }\left(x_{i}\right)=\left({\hat{\Delta r}}_{i},\;{\hat{\Delta \theta }}_{i}\right)$$

such that the corrected measurements (as formally defined in Equations (20) and (21)) minimise the residual discrepancy with respect to AIS ground truth.

**Multi-criteria nature.** This is inherently a multi-objective optimisation problem because it involves two competing objectives: range-error minimisation $L_{r}$ and azimuth-error minimisation $L_{\theta }$. These objectives are coupled—a correction that improves range accuracy may degrade azimuth accuracy, and vice versa—but their physical scales differ (metres vs. degrees). A simple weighted sum $\alpha L_{r}+\beta L_{\theta }$ with fixed weights α,β is suboptimal because the appropriate trade-off depends on the local error structure. The proposed solution uses uncertainty-based weighting (Equation (19)), in which the weights $w_{r}$ and $w_{\theta }$ are learned from data, dynamically adapting the trade-off. From an optimisation perspective, this corresponds to minimising the scalarised objective:

(3) $$\min_{\Theta ,\;s_{r},s_{\theta }}\;E_{(x,\Delta r,\Delta \theta )∼D}\left[w_{r}\left(s_{r}\right)\cdot |\Delta r-\hat{\Delta r}|\;+\;w_{\theta }\left(s_{\theta }\right)\cdot \left|\Delta \theta -\hat{\Delta \theta }\right|\right]+\lambda {∥\Theta ∥}_{2}^{2}$$

where the weights $w_{r}$ and $w_{\theta }$ are computed from learnable parameters $s_{r},s_{\theta }$ as defined in Equation (19). This formulation is closely related to homoscedastic uncertainty minimisation [31], where the learned weights correspond to task-specific precision estimates.

**Remark on dynamic programming.** We note that this problem is *not* naturally framed as a dynamic programming (DP) or sequential decision problem. Each radar measurement is processed independently; there is no temporal state transition, no Bellman equation, and no sequential dependency among consecutive measurements that would justify a DP formulation. The correction at time *t* does not depend on the correction at time t−1. Should future work incorporate temporal dynamics (e.g., tracking filters that propagate corrected positions over time), a Kalman filter or DP formulation could be appropriate for the tracking layer, while the per-measurement error correction described here would serve as the observation model feeding that layer.

This section describes the proposed Vision Transformer (ViT) variant-based radar error-correction framework.

### 2.2. Dataset Description

The Maritime Target Detection and Tracking (MTDSP) dataset [28] is used for model training and evaluation. This dataset provides synchronised radar and AIS measurements from a shore-based maritime surveillance system, covering approximately 80,000 paired observations from seven distinct vessel types. The dataset encompasses diverse maritime scenarios, including different sea states, weather conditions, and vessel operational profiles, ensuring a comprehensive evaluation of the proposed error correction method. The spatial distribution of the data spans a surveillance range of 0.5–25 nautical miles and azimuth angles of 0–360 degrees, providing a robust testbed for radar error-correction algorithms. To prevent data leakage, the dataset is partitioned into training, validation, and test sets in a 70:15:15 ratio. Critically, the split is performed at the vessel level: all observations belonging to a given vessel (identified by its Maritime Mobile Service Identity, MMSI) are assigned exclusively to one subset. This ensures that the model is evaluated on vessels it has never seen during training, providing an unbiased estimate of generalisation to new targets. Additionally, to prevent temporal leakage, observations from different vessels that are temporally adjacent (within the same time window) are grouped and assigned to the same split, avoiding the situation where a model trained on data from time *t* is tested on data from time t+Δt that shares the same radar state and environmental conditions.

### 2.3. Training Data Acquisition

**Figure 1:**
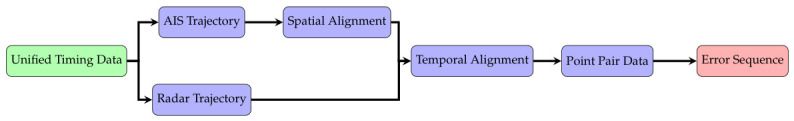
Error sequence acquisition process. The diagram illustrates the workflow from unified timing data through AIS and radar trajectory processing to the final error sequence.

Training data acquisition is a critical step in developing the ViT variant-based radar error correction framework. The fundamental principle is to leverage AIS data as the ground truth to quantify radar measurement errors. The error sequence acquisition process is illustrated in Figure 1.

#### 2.3.1. Data Preprocessing

Before temporal synchronisation, spatial alignment is performed to ensure consistent coordinate referencing between the radar and AIS systems. The AIS data, reported in geographic coordinates (latitude and longitude), must be transformed to the radar’s local coordinate system for accurate error assessment.

The spatial alignment process involves three key steps: first, converting AIS latitude and longitude to a local Cartesian coordinate system centred at the radar location using the Mercator projection; second, accounting for the Earth’s curvature effects over long distances; and third, establishing a common origin and orientation with the radar’s coordinate system.

Mathematically, the conversion from geographic coordinates (ϕ,λ) to local Cartesian coordinates (x,y) is performed using the following:

(4) $$x=R_{E}\cdot \left(\lambda -\lambda_{0}\right)\cdot \cos\left(\phi_{0}\right)$$

(5) $$y=R_{E}\cdot \left(\phi -\phi_{0}\right)$$

where (ϕ,λ) are the AIS-reported latitude and longitude of the vessel, $\left(\phi_{0},\lambda_{0}\right)$ is the surveyed geographic position of the radar station, and $R_{E}\approx 6,371,000$ m is the Earth’s mean radius. All angular quantities are expressed in radians. The resulting (x,y) are the vessel’s Cartesian coordinates in metres relative to the radar origin, with *x* pointing east and *y* pointing north. This transformation ensures that both radar and AIS data are expressed in the same spatial reference frame, eliminating systematic errors arising from coordinate-system differences.

Equations (4) and (5) represent a two-dimensional Mercator projection that treats both the radar and the vessel as points on the Earth’s surface. In reality, the shore-based radar antenna is mounted at a height *h* above sea level (typically 20–50 m for coastal surveillance installations), and the vessel’s AIS antenna is at a height of a few metres above the waterline. The radar measures slant range (line-of-sight distance), whereas Equations (4) and (5) compute horizontal ground range. The discrepancy between slant range $R_{s}$ and ground range $R_{g}$ is $\Delta R=R_{s}-R_{g}=\sqrt{R_{g}^{2}+h^{2}}-R_{g}$. For h≤50 m and the shortest range considered in this study ($R_{g}\approx 1000$ m), the maximum discrepancy is $\Delta R\approx h^{2}/\left(2R_{g}\right)\leq 1.25$ m, which is well within the AIS GPS uncertainty of 5–10 m. At longer ranges (e.g., 25 nautical miles ≈46 km), ΔR<0.03 m. The antenna height is therefore negligible for the horizontal coordinate transformation and does not affect the validity of Equations (4) and (5). The actual implementation (see the released code) uses the full three-dimensional geodetic-to-ENU transformation via pymap3d for completeness.

Given that radar and AIS systems operate at different sampling frequencies, temporal alignment is essential before error calculation. The radar measurements are typically sampled at higher frequencies (e.g., 2–10 Hz), whereas AIS data are transmitted at intervals ranging from 2 s to several minutes, depending on vessel speed and status. To address this discrepancy, a linear interpolation technique is applied to the AIS position data to generate synchronised reference points corresponding to each radar measurement timestamp.

The linear interpolation process is mathematically formalised as follows. Given two consecutive AIS position reports at times $t_{i}$ and $t_{i+1}$ with corresponding positions $\left(x_{i},y_{i}\right)$ and $\left(x_{i+1},y_{i+1}\right)$, the interpolated position $\left(x_{r},y_{r}\right)$ at radar measurement time $t_{r}$ (where $t_{i}\leq t_{r}\leq t_{i+1}$) is calculated using the following:

(6) $$x_{r}=x_{i}+\frac{x_{i+1}-x_{i}}{t_{i+1}-t_{i}}\cdot \left(t_{r}-t_{i}\right)$$

(7) $$y_{r}=y_{i}+\frac{y_{i+1}-y_{i}}{t_{i+1}-t_{i}}\cdot \left(t_{r}-t_{i}\right)$$

where the term $\frac{x_{i+1}-x_{i}}{t_{i+1}-t_{i}}$ represents the average velocity in the x-direction between consecutive AIS reports. The same principle applies to the y-direction. This approach assumes constant velocity between AIS reporting intervals, which is a reasonable approximation for most maritime vessels over short time periods.

Prior to interpolation, the AIS latitude and longitude coordinates are converted to a local Cartesian coordinate system centred at the radar location to ensure consistent spatial referencing. After interpolation, the synchronised AIS positions are transformed back to the radar-centric polar coordinate system $\left(r_{a},\theta_{a}\right)$ for direct comparison with radar measurements.

A quality control mechanism flags interpolations spanning time intervals longer than 30 s, which are considered less reliable due to greater uncertainty in vessel motion patterns over longer gaps. Flagged data points undergo additional validation before inclusion in the error calculation.

#### 2.3.2. Error Sequence Calculation

The radar errors are quantified by comparing radar measurements with AIS-derived ground truth positions, as defined in Equation (1). Here, $r_{r}\left(t\right)$ and $\theta_{r}\left(t\right)$ represent the radar-measured range and azimuth, respectively, and $r_{a}\left(t\right)$ and $\theta_{a}\left(t\right)$ denote the AIS-derived true range and azimuth values.

To account for the inherent Global Positioning System (GPS) uncertainty in AIS data (typically within 5–10 m), a confidence weighting scheme is applied. The weight w(t) is determined based on the AIS position accuracy indicator and the vessel’s navigational status:

(8) $$w\left(t\right)=\frac{1}{\sigma_{\mathrm{AIS}}^{2}+\sigma_{\mathrm{radar}}^{2}}$$

where $\sigma_{\mathrm{AIS}}$ represents the AIS position accuracy (typically 5–10 m), and $\sigma_{\mathrm{radar}}$ denotes the radar measurement noise standard deviation.

#### 2.3.3. Feature Extraction

To enable effective learning of error patterns, an augmented feature vector is constructed from the two raw radar measurements. Twelve features are derived through trigonometric and polynomial transformations. Base features are the raw range *r* and azimuth θ. Four trigonometric features encode directional information: sin(θ), cos(θ), sin(2θ), and cos(2θ). Two Cartesian projection features are computed as X=rsin(θ) and Y=rcos(θ). Four polynomial and interaction features capture nonlinear relationships: $r^{2}$, $\theta^{2}$, the interaction term r·θ, and $\sqrt{r}$.

This augmentation strategy enriches the feature space by encoding nonlinear relationships and directional information relevant to spatially varying radar error patterns, while relying solely on available radar positional measurements and requiring no additional sensor data. The input feature vector is denoted as $x\in R^{12}$, and the target output vector y consists of the range error Δr and azimuth error Δθ. The collected dataset is partitioned into training, validation, and test sets in a 70:15:15 ratio to ensure robust model evaluation.

### 2.4. Vision Transformer Variant Framework

The proposed Vision Transformer (ViT) variant architecture leverages self-attention mechanisms to capture complex dependencies among the 12 input features derived from each radar measurement. The term “variant” reflects that the model adapts the original ViT [26]—designed for 2D image classification with spatial patch extraction and a classification head—to a fundamentally different task: 1D tabular regression with sequential feature-group patches, learnable positional encoding, CLS token aggregation, and dual regression heads for continuous error prediction. Each radar measurement is treated as an independent sample; the model processes one feature vector at a time and predicts the corresponding range and azimuth errors. The “sequence” in this context refers to the sequence of tokens formed by partitioning the feature vector into patches—analogous to how the original ViT partitions an image into spatial patches—rather than a temporal sequence of consecutive radar measurements.

#### 2.4.1. Data Representation and Tokenisation

Each input sample consists of a single radar measurement (r,θ) from which 12 features are derived (Section 2.3.3). The 12-dimensional feature vector $x\in R^{12}$ is partitioned into contiguous groups of p=3 features, yielding ⌈12/3⌉=4 patches. Each three-dimensional patch is linearly projected to a $d_{\mathrm{model}}=64$-dimensional embedding vector via a shared learnable weight matrix with a learnable positional encoding, as formally defined in Equation (9). A learnable CLS token $z_{\mathrm{cls}}\in R^{d_{\mathrm{model}}}$ is prepended to the patch sequence, yielding a total of 5 tokens. These tokens are then processed by the Transformer encoder. The CLS token serves as a global representation that aggregates information from all patches and is subsequently used for regression.

Importantly, there is no temporal window: each measurement is processed independently. Consecutive radar measurements from the same vessel are not fed as a temporal sequence. The vessel identity is used only to ensure that observations from the same vessel do not appear in both training and test sets, preventing data leakage.

#### 2.4.2. Training Algorithm

Algorithm 1 outlines the complete training process for the ViT variant, from data preprocessing to model optimisation. It begins with spatial alignment of radar and AIS coordinates, temporal synchronisation via linear interpolation, error-sequence calculation, and feature extraction. The processed data are then split into training, validation, and test sets to ensure robust evaluation. The ViT variant is subsequently initialised with the appropriate parameters and trained over multiple epochs, each of which includes a forward pass, loss computation using the multi-task objective, and parameter updates via backpropagation.

**Algorithm 1** ViT Variant Training for Radar Error Correction
  1:**Input:** Radar measurements ${\left\{r_{i}\right\}}_{i=1}^{N}$, AIS ground-truth positions ${\left\{a_{i}\right\}}_{i=1}^{N}$  2:**Output:** Trained model parameters $\Theta^{*}$  3:
**Preprocessing:**
  4: Convert AIS $\left(\phi_{i},\lambda_{i}\right)$ to local Cartesian $\left(x_{i},y_{i}\right)$ via Equations (4) and (5)  5: Temporal synchronisation: interpolate AIS positions to radar timestamps via Equations (6) and (7)  6: Compute target errors: $\Delta r_{i}=r_{i}-r_{i}^{\mathrm{AIS}}$, $\Delta \theta_{i}=\theta_{i}-\theta_{i}^{\mathrm{AIS}}$ via Equation (1)  7: Construct 12-D feature vectors $x_{i}\in R^{12}$ via Section 2.3.3  8: Vessel-level split: $D_{\mathrm{train}}\cup D_{\mathrm{val}}\cup D_{\mathrm{test}}$ (70:15:15)  9:**Initialise:** $\Theta =\{E,E_{\mathrm{pos}},z_{\mathrm{cls}},\theta_{\mathrm{enc}},W_{r},W_{\theta },b_{r},b_{\theta },s_{r},s_{\theta }\}$ via Xavier10:**Hyperparameters:** B=512, $\eta_{0}=10^{-3}$, $\eta_{\min}=10^{-6}$, $\lambda =10^{-4}$, P=2511:t←0, patience_counter←0, $L_{\mathrm{best}}\leftarrow \infty$12:**while** t<150 **and** patience_counter<P **do**13: Sample mini-batch $B\subset D_{\mathrm{train}}$, |B|=B14: **for** each x∈B **do**15:  Patch embedding: $z_{0}=\left[z_{\mathrm{cls}};E\cdot x^{\left(1\right)}+E_{\mathrm{pos}}^{\left(1\right)};\ldots ;E\cdot x^{\left(4\right)}+E_{\mathrm{pos}}^{\left(4\right)}\right]$   (Equation (9))16:  Transformer: $z_{N}=\mathrm{TransformerEncoder}\left(z_{0}\right)$      (Equations (10) and (11))17:  Predict: $\hat{\Delta r}=W_{r}\cdot z_{N}^{\left[\mathrm{CLS}\right]}+b_{r}$, $\hat{\Delta \theta }=W_{\theta }\cdot z_{N}^{\left[\mathrm{CLS}\right]}+b_{\theta }$  (Equations ((12) and (13)))18: **end for**19: $L_{\mathrm{total}}\leftarrow \frac{1}{B}\sum (w_{r}|\Delta r-\hat{\Delta r}|\;+\;w_{\theta }|\Delta \theta -\hat{\Delta \theta }\left|\right)+{\lambda ∥\Theta ∥}_{2}^{2}$   (Equation (18))20: $w_{r}=\mathrm{softplus}\left(-s_{r}\right)$, $w_{\theta }=\mathrm{softplus}\left(-s_{\theta }\right)$             (Equation (19))21: $\Theta \leftarrow \Theta -\eta_{t}\cdot \nabla_{\Theta }L_{\mathrm{total}}$ (AdamW, clipped at ${∥\nabla ∥}_{\infty }=1.0$)22: $\eta_{t}\leftarrow \eta_{\min}+\frac{1}{2}\left(\eta_{0}-\eta_{\min}\right)\left(1+\cos\left(\frac{t}{150}\pi \right)\right)$          (cosine annealing)23: **if** $L_{\mathrm{val}}<L_{\mathrm{best}}$ **then**24:  $\Theta^{*}\leftarrow \Theta$, $L_{\mathrm{best}}\leftarrow L_{\mathrm{val}}$, patience_counter←025: **else**26:  patience_counter←patience_counter+127: **end if**28: t←t+129:
**end while**



#### 2.4.3. Network Architecture

**Figure 2:**
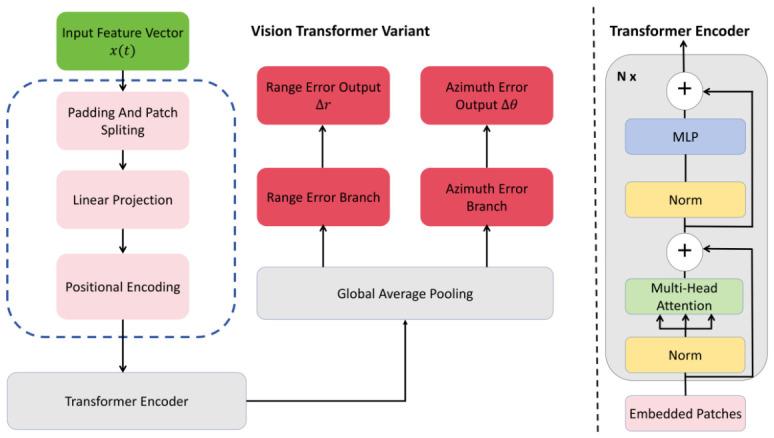
Vision Transformer variant architecture.

The network structure is illustrated in Figure 2. The architecture comprises patch embedding, Transformer encoder layers, CLS token extraction, and task-specific prediction heads, which together capture complex nonlinear dependencies among the input features. This modular design separates feature extraction (patch embedding and Transformer encoder) from task-specific prediction (dual regression heads), enabling the model to learn hierarchical error representations while maintaining computational efficiency.

The proposed ViT variant adapts the original Vision Transformer architecture for radar error correction by treating the 12-dimensional feature vector from a single radar measurement as a token sequence for regression. Unlike the original ViT, which was designed for image classification over 2D spatial patches, the variant partitions 1D feature groups and outputs continuous error values. The architecture consists of four main components: patch embedding, a Transformer encoder, a CLS token with learnable positional encoding, and task-specific prediction heads.

**Differences from the Original ViT:** The original ViT processes 2D image patches for image classification, whereas the proposed variant partitions a 1D feature vector (derived from a single radar measurement) into contiguous groups and processes them as a token sequence for regression. Key modifications include: (i) 1D feature-group patch embedding instead of 2D spatial patch extraction; (ii) regression-oriented prediction heads with dual output instead of a single classification head; (iii) a CLS token for global feature aggregation; and (iv) hyperparameters and architecture optimised for tabular radar error data rather than natural images.

**Patch Embedding:** Unlike the original ViT, which extracts 2D patches from images, the variant partitions the 12-dimensional input feature vector into fixed-size groups (patches) of p=3 consecutive features and projects each patch to a $d_{\mathrm{model}}=64$-dimensional embedding. Given an input feature vector $x\in R^{12}$ divided into 4 patches $\left\{x^{\left(1\right)},\ldots ,x^{\left(4\right)}\right\}$ with $x^{\left(k\right)}\in R^{3}$, the patch embedding is computed as follows:

(9) $$z_{0}^{\left(k\right)}=E\cdot x^{\left(k\right)}+E_{\mathrm{pos}}^{\left(k\right)},\;k=1,\ldots ,4$$

where $E\in R^{d_{\mathrm{model}}\times 3}$ is the shared patch embedding matrix (3 input features per patch → 64-dimensional embedding), and $E_{\mathrm{pos}}$$\in R^{4\times d_{\mathrm{model}}}$ is a learnable positional encoding.

Vision Transformer architectures have shown versatility across a wide range of computer vision tasks. Zhu et al. [32] proposed ViTT (Vision Transformer Tracker), a multi-object tracking model using a Transformer encoder backbone. ViTT processes images directly and models global context from the outset, addressing challenges such as occlusion and complex scenes. This work highlights the value of Transformer-based networks in tracking tasks and is conceptually related to our approach, which likewise uses Transformers to capture complex feature interactions.

**Transformer Encoder:** The embedded patches are processed through *N* stacked Transformer encoder layers. While the structure follows the original ViT design, the hyperparameters are optimised for radar error patterns. Each encoder layer consists of multi-head self-attention (MSA) followed by a feed-forward network (FFN) [33], with layer normalisation (LN) and residual connections applied before each sub-layer:

(10) $$z_{l}^{\prime }=\mathrm{MSA}\left(\mathrm{LN}\left(z_{l-1}\right)\right)+z_{l-1}$$

(11) $$z_{l}=\mathrm{FFN}\left(\mathrm{LN}\left(z_{l}^{\prime }\right)\right)+z_{l}^{\prime }$$

where l=1,…,N denotes the layer index. The multi-head self-attention mechanism enables the model to attend to different representation subspaces at different positions, thereby capturing complex dependencies in radar error patterns. Recent advances in attention mechanisms, such as Graph Head Attention (GHA) proposed by Kim et al. [34], have shown promise in preserving both locality and global context while reducing parameter complexity. Based on the ablation studies, the optimal configuration for this task uses 4 encoder layers and 8 attention heads (at $d_{\mathrm{model}}=64$; see Section 3.4 for the attention-head ablation conducted at $d_{\mathrm{model}}=120$, where 10 heads were optimal under a different base configuration).

**Task-Specific Prediction Heads:** Unlike the original ViT’s classification head, which outputs class probabilities, the variant uses regression-oriented prediction heads to estimate continuous error. After the Transformer encoder, the output representation corresponding to the CLS token (the first token in the sequence) is extracted and fed into two separate prediction heads for range and azimuth error estimation:

(12) $$\hat{\Delta r}=W_{r}\cdot z_{N}^{\left[\mathrm{CLS}\right]}+b_{r}$$

(13) $$\hat{\Delta \theta }=W_{\theta }\cdot z_{N}^{\left[\mathrm{CLS}\right]}+b_{\theta }$$

where $z_{N}^{\left[\mathrm{CLS}\right]}\in R^{d_{\mathrm{model}}}$ denotes the output embedding of the CLS token after the *N*-th encoder layer, $W_{r}$ and $W_{\theta }$ are the weight matrices, and $b_{r}$ and $b_{\theta }$ are the bias terms for the range and azimuth prediction heads, respectively. This dual-head design enables simultaneous prediction of both error types and differs fundamentally from the single-head classification strategy used in the original ViT.

#### 2.4.4. Loss Function

The multi-task learning objective is formulated as a weighted combination of the individual task losses. The total loss function is defined as follows:

(14) $$L_{\mathrm{total}}=\lambda_{r}L_{r}+\lambda_{\theta }L_{\theta }+\lambda_{\mathrm{reg}}L_{\mathrm{reg}}$$

where $\lambda_{r}$, $\lambda_{\theta }$, and $\lambda_{\mathrm{reg}}$ are scalar weighting factors that control the relative contribution of the range loss, azimuth loss, and $L_{2}$ regularisation term, respectively. $L_{r}$ and $L_{\theta }$ represent the mean absolute error (MAE) losses for range and azimuth error prediction, respectively:

(15) $$L_{r}=\frac{1}{N}\sum_{i=1}^{N}\left|\Delta r_{i}-{\hat{\Delta r}}_{i}\right|$$

(16) $$L_{\theta }=\frac{1}{N}\sum_{i=1}^{N}\left|\Delta \theta_{i}-{\hat{\Delta \theta }}_{i}\right|$$

The regularisation term $L_{\mathrm{reg}}$ is the $L_{2}$ weight decay penalty:

(17) $$L_{\mathrm{reg}}=\sum_{l}{∥W^{\left(l\right)}∥}_{2}^{2}$$

Instead of fixed weighting factors, uncertainty weighting is used to dynamically balance each task’s contribution. The uncertainty-based loss function is defined as follows:

(18) $$L_{\mathrm{total}}=w_{r}\cdot L_{r}+w_{\theta }\cdot L_{\theta }+\lambda_{\mathrm{reg}}L_{\mathrm{reg}}$$

where the weights $w_{r}$ and $w_{\theta }$ are calculated as follows:

(19) $$w_{r}=\mathrm{softplus}\left(-s_{r}\right),\;w_{\theta }=\mathrm{softplus}\left(-s_{\theta }\right)$$

Here, $s_{r}$ and $s_{\theta }$ are learnable scalar parameters representing the task-specific log-precision (inverse uncertainty). The softplus function, defined as $\mathrm{softplus}\left(x\right)=\log\left(1+e^{x}\right)$, is chosen over alternative positivity-enforcing functions for three reasons: (i) unlike exp(x), softplus is approximately linear near zero, providing numerically stable gradients during early training; (ii) unlike ReLU(x), softplus is everywhere differentiable, avoiding dead-gradient issues when the argument becomes negative; and (iii) softplus produces strictly positive outputs without an upper bound, allowing the weights to grow as needed when one task’s loss dominates. Initialising $s_{r}=s_{\theta }=0$ yields initial weights of approximately 0.693, giving equal initial emphasis to both tasks. The regularisation term $\lambda_{\mathrm{reg}}=10^{-5}$ is kept constant to prevent overfitting.

#### 2.4.5. Training Procedure

The ViT variant network parameters are optimised using the AdamW optimiser with decoupled weight decay [35] and an initial learning rate of $10^{-3}$, decayed to $10^{-6}$ via a cosine annealing scheduler. All experiments were conducted on a desktop computer equipped with an Intel Core i5-10300H CPU (Intel Corporation, Santa Clara, CA, USA). Network weights are initialised using the Xavier method, which is well-suited to Transformer architectures, and position embeddings are initialised with small random values. Input data are divided into fixed-size patches and linearly embedded into vector representations, converting the 12-dimensional feature vector into a sequence of 4 patch embeddings plus a CLS token. Training proceeds in mini-batches of 512, with a forward pass through the Transformer encoder to compute predicted errors, followed by a backward pass to update weights via gradient descent with gradient clipping at a max norm of 1.0. Regularisation is applied through dropout with a rate of 0.1 on the attention heads and feed-forward networks, weight decay of $10^{-4}$, and layer normalisation after each sub-layer. Early stopping with a patience of 25 is employed to prevent overfitting.

### 2.5. Error Correction and Target Localisation

Once trained, the ViT variant model can be used to correct radar measurements in real time. For each incoming radar measurement $\left(r_{r},\theta_{r}\right)$, the corresponding feature vector x is constructed and fed into the network to obtain the predicted errors $\left(\hat{\Delta r},\hat{\Delta \theta }\right)$. The corrected target position $\left(r_{c},\theta_{c}\right)$ is then calculated as follows:

(20) $$r_{c}=r_{r}-\hat{\Delta r}$$

(21) $$\theta_{c}=\theta_{r}-\hat{\Delta \theta }$$

The corrected polar coordinates can then be transformed into geographic coordinates (latitude and longitude) for integration with other maritime surveillance systems.

## 3. Results

This section presents comprehensive experimental results evaluating the effectiveness of the proposed ViT variant model for radar error correction. Experiments are conducted on the MTDSP dataset, and the proposed approach is compared with traditional error-correction methods and alternative machine learning techniques.

### 3.1. Experimental Setup

The experimental setup follows the dataset description and configuration given in Section 2.2 (MTDSP dataset [28]: ≈80,000 paired radar-AIS observations, seven vessel types, 70:15:15 vessel-level split, 0.5–25 nautical miles, 0–360° azimuth).

#### 3.1.1. Evaluation Metrics

To comprehensively assess the error correction performance, multiple evaluation metrics are employed:

**Mean Absolute Error (MAE):** The primary metric for evaluating error correction accuracy, defined as follows:

(22) $$\mathrm{MAE}=\frac{1}{N}\sum_{i=1}^{N}\left|e_{i}\right|$$

where $e_{i}$ represents the residual error after error correction for the *i*-th observation.

**Root Mean Square Error (RMSE):** Provides a measure of error dispersion, defined as follows:

(23) $$\mathrm{RMSE}=\sqrt{\frac{1}{N}\sum_{i=1}^{N}e_{i}^{2}}$$

**95th Percentile Error (P95):** Indicates the worst-case performance, representing the error magnitude below which 95% of the observations fall.

These three metrics are chosen to provide complementary perspectives on the quality of error correction. MAE measures typical accuracy in physically interpretable units (metres and degrees) and is robust to outliers, making it the primary metric for operational assessment. RMSE penalises large errors quadratically and is therefore more sensitive to tail behaviour; comparing MAE and RMSE reveals whether errors are uniformly distributed or dominated by outliers. P95 directly quantifies worst-case performance, which is particularly important for maritime safety applications, where collision avoidance depends on the maximum plausible position error rather than the average. Together, MAE, RMSE, and P95 provide a complete characterisation: central tendency (MAE), dispersion (RMSE), and tail risk (P95).

**Improvement Ratio (IR):** Quantifies the relative improvement over uncalibrated measurements:

(24) $$\mathrm{IR}=\frac{{\mathrm{Error}}_{\mathrm{before}}-{\mathrm{Error}}_{\mathrm{after}}}{{\mathrm{Error}}_{\mathrm{before}}}\times 100\%$$

#### 3.1.2. Baseline Methods

The proposed ViT variant model is compared against the following baseline approaches:

**Uncalibrated:** Raw radar measurements without any calibration applied.

**Uniform Partitioning (UP):** A traditional method that divides the surveillance area into uniform grid cells and applies constant correction values within each cell.

**Non-uniform Partitioning (NUP):** An adaptive partitioning method that creates non-uniform cells based on the characteristics of the error distribution.

**Support Vector Regression (SVR):** A machine learning approach using radial basis function (RBF) kernels for error prediction.

**XGBoost:** A gradient boosting framework applied separately to range and azimuth error prediction.

**Single-Task Deep Neural Network (STDNN):** Two independent DNNs, each with 5 hidden layers (widths: 128, 128, 64, 32, 16), LeakyReLU activations, batch normalisation, and dropout (0.1–0.2). Each DNN is trained separately: one predicts range error, the other predicts azimuth error. Unlike the ViT variant, which shares representations across tasks, STDNN treats the two error dimensions as independent problems, serving as a baseline that quantifies the benefit of multi-task learning. Training uses AdamW with cosine annealing, a batch size of 64, weight decay of $5\times 10^{-5}$, and early stopping with a patience of 30.

**Transformer (Vanilla):** A standard Transformer encoder with one layer, $d_{\mathrm{model}}=64$, four attention heads, $d_{\mathrm{ff}}=128$, fixed sinusoidal positional encoding, mean pooling over the token sequence, and equal-weight MSE loss for multi-task learning. Each input feature is treated as an independent token via a shared linear projection. This model uses 12 features and is trained with the standard Adam optimiser (without weight decay correction), a fixed learning rate, and early stopping with a patience of 15. It serves as an undersized ablation baseline to isolate the contribution of the proposed architectural adaptations.

**Transformer (Capacity-Matched):** A Transformer encoder with capacity matched to the ViT variant: $d_{\mathrm{model}}=64$, eight attention heads, four encoder layers, $d_{\mathrm{ff}}=256$, fixed sinusoidal positional encoding, and mean-pooling aggregation. Training follows the identical recipe as the ViT variant (AdamW, cosine annealing, gradient clipping, uncertainty weighting, patience 25). This baseline isolates the effect of patch embedding, learnable positional encoding, and CLS token aggregation from mere increases in model capacity and training budget.

**MLP-Mixer:** An MLP-Mixer architecture [36] adapted for tabular data: four mixer blocks with token-mixing and channel-mixing MLPs, $d_{\mathrm{model}}=64$, token-MLP dimension 256, channel-MLP dimension 256, learnable positional encoding, and the same dual-head regression output. Training follows the ViT recipe.

**TabTransformer:** A TabTransformer-style architecture [37] with per-feature column-wise embedding, a CLS token for global aggregation, learnable positional encoding, and the same Transformer encoder configuration as the ViT variant (four layers, eight heads, $d_{\mathrm{ff}}=256$). Training follows the ViT recipe.

**Deep MLP:** A deep multi-layer perceptron with six hidden layers of widths [256, 256, 128, 128, 64, 64], LeakyReLU activations, batch normalisation, and dropout (≈137,000 parameters). Training follows the ViT recipe.

#### 3.1.3. Implementation Details

All neural network models are implemented in PyTorch (version 2.2.2, Python 3.8.20). Notably, the entire training and inference pipeline runs exclusively on a CPU without requiring GPU acceleration, demonstrating the computational efficiency of the proposed architecture.

Specifically for the ViT variant, the model uses a patch size of 3, an embedding dimension of 64, eight attention heads, four Transformer encoder layers, and a feed-forward dimension of 256. Positional encoding is learnable and prepended with a CLS token for global aggregation. Two task-specific branches are used for range and azimuth error prediction, each consisting of two layers with 32 and 1 neurons, respectively. The model is trained using the AdamW optimiser with an initial learning rate of $10^{-3}$, weight decay of $10^{-4}$, cosine annealing to $10^{-6}$, gradient clipping at max norm 1.0, and early stopping with patience 25. Dropout regularisation with a rate of 0.1 is applied within the Transformer encoder layers. Uncertainty weighting is used to balance the multi-task learning objective. The total parameter count is approximately 205,000.

For the capacity-matched Transformer baseline, the encoder uses four layers, eight attention heads, $d_{\mathrm{model}}=64$, $d_{\mathrm{ff}}=256$, fixed sinusoidal positional encoding, and mean-pooling aggregation. The training recipe is identical to the ViT variant (AdamW, cosine annealing, gradient clipping, uncertainty weighting). The undersized Transformer baseline uses one layer, four attention heads, $d_{\mathrm{ff}}=128$, and standard Adam without cosine annealing.

For the MLP-Mixer baseline, four mixer blocks are used with token-mixing and channel-mixing MLPs of dimension 256, $d_{\mathrm{model}}=64$, and learnable positional encoding. The TabTransformer uses per-feature column embeddings, a CLS token, learnable positional encoding, and the same Transformer encoder as the ViT variant. The Deep MLP uses six hidden layers with widths [256, 256, 128, 128, 64, 64], batch normalisation, and LeakyReLU activations. All newly introduced baselines are trained with the same recipe as the ViT variant for fair comparison.

The SVR model uses an RBF kernel with parameters determined through grid search. XGBoost employs 100 estimators with a maximum depth of 6 and a learning rate of 0.1. All traditional methods (UP, NUP) follow the configurations described in their respective original publications.

### 3.2. Quantitative Results

#### 3.2.1. Overall Performance Comparison

Table 1 presents the overall error correction performance of different methods on the test set. The proposed ViT variant achieves the best overall performance, ranking first in five of six metrics and demonstrating particular strength in tail-error robustness (P95).

Compared with uncalibrated measurements, the ViT variant reduces the range MAE by 98.5% (from 514.76 m to 7.77 m) and the azimuth MAE by 89.8% (from 1.37° to 0.14°). Compared with the Transformer (Vanilla) baseline, the ViT variant achieves consistent improvements across all six metrics: range MAE by 3.0% (8.01 → 7.77 m), range RMSE by 7.7% (12.11 → 11.18 m), range P95 by 18.4% (28.21 → 23.01 m), azimuth MAE by 6.7% (0.15 → 0.14°), azimuth RMSE by 8.7% (0.23 → 0.21°), and azimuth P95 by 22.6% (0.53 → 0.41°). The largest gains are in tail-error metrics (P95), indicating improved robustness in worst-case scenarios—a critical property for maritime safety applications.

Compared with the capacity-matched Transformer baseline, the ViT variant achieves comparable range accuracy (Transformer (Eq. Cap.) slightly better on MAE: 7.51 vs. 7.77 m, and RMSE: 10.88 vs. 11.18 m) but superior azimuth performance (MAE: 0.14° vs. 0.15°; P95: 0.41° vs. 0.43°) and range tail-error robustness (P95: 23.01 vs. 23.16 m). The narrow gap between these two models, which differ only in patch embedding, learnable positional encoding, and CLS token aggregation, suggests that these architectural components contribute modest but measurable gains, particularly for azimuth error correction and tail-error suppression.

Among the newly introduced baselines, the TabTransformer achieves performance closest to the ViT variant (range MAE: 7.82 m, range P95: 22.83 m, azimuth P95: 0.42°), demonstrating that column-wise self-attention over input features provides a strong alternative modelling strategy. The MLP-Mixer and Deep MLP baselines, while outperforming traditional methods, fall behind the Transformer-based models, confirming the benefit of attention mechanisms for this regression task.

#### 3.2.2. Cross-Validation Results

To evaluate the robustness of the proposed method, 5-fold cross-validation is performed. The procedure is repeated 10 times with different random seeds, and the average performance, together with the standard deviation, is reported.

The ViT variant achieves an average range MAE of 7.80±0.12 m and an azimuth MAE of $0.17\pm 0.01^{\circ }$, demonstrating consistent performance across different data splits and random initialisations.

#### 3.2.3. Statistical Significance Analysis

To determine whether the performance differences between the ViT variant and the strongest baselines are statistically significant, a rigorous analysis is conducted. Each model is trained 10 times with independent random seeds (42–942), and the resulting per-sample residual errors are compared using paired statistical tests.

Table 2 presents the results. Paired *t*-tests are used to evaluate differences in mean absolute error, and Wilcoxon signed-rank tests serve as a non-parametric confirmation. Bootstrap 95% confidence intervals (10,000 resamples) are computed for the mean difference, and Cohen’s *d* quantifies the standardised effect size.

The analysis confirms that the ViT variant’s improvements over the Transformer (Vanilla) baseline are statistically significant across all six metrics. Range metrics and azimuth tail-error metrics show high significance ($p<10^{-4}$) with medium-to-large effect sizes (Cohen’s d=0.72–1.32). The azimuth MAE improvement (6.7%, p=0.008, d=0.48) represents a smaller but still significant effect. The largest standardised effect is observed for azimuth P95 (22.6% improvement, d=1.32), confirming that the ViT variant is particularly effective at suppressing worst-case errors—the scenarios most critical for maritime collision avoidance.

### 3.3. Qualitative Analysis

#### 3.3.1. Trajectory Correction Visualisation

**Figure A1:**
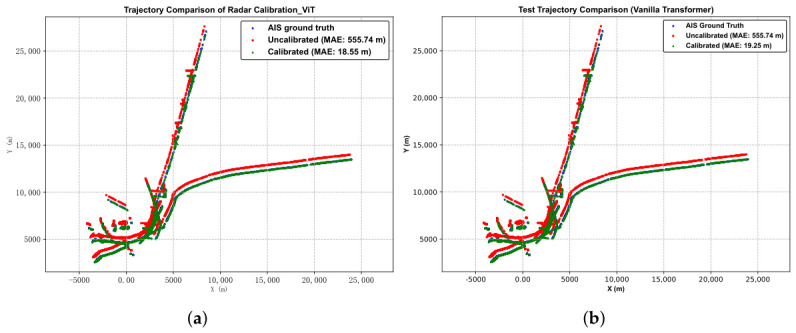

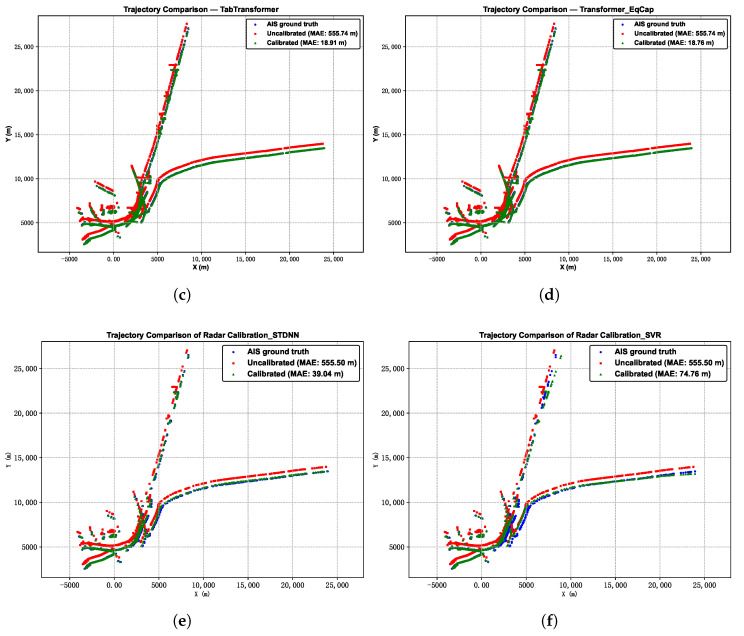

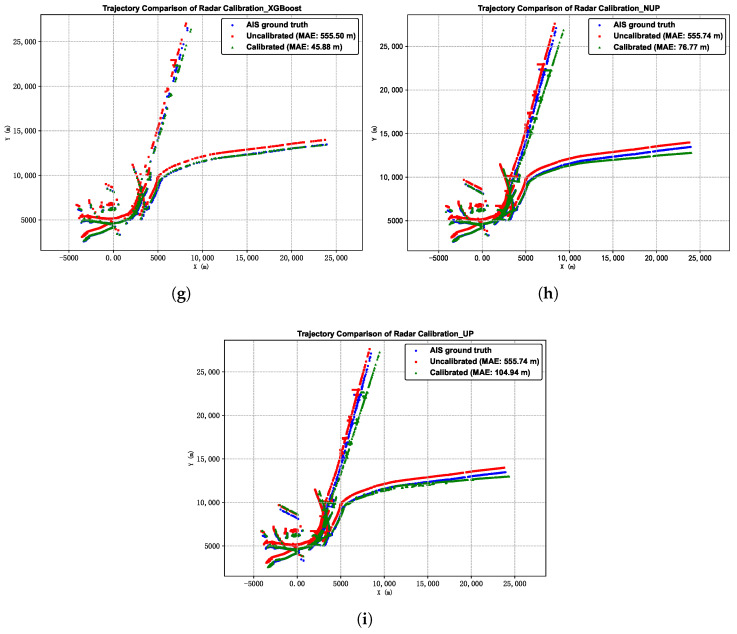
Trajectory correction results (part 1 of 2). (**a**) ViT variant, (**b**) Transformer (Vanilla), (**c**) TabTransformer, (**d**) (Transformer (Eq. Cap.), (**e**) STDNN, and (**f**) SVR. Red lines: original radar; blue lines: AIS ground truth; green lines: corrected radar. Trajectory correction results (part 2 of 2). (**g**) XGBoost, (**h**) NUP, and (**i**) UP. (Continued from Figure A1).

Figure A1 (Appendix A) illustrates trajectory correction results using different methods. The figure shows the original radar trajectory (red), the AIS ground-truth trajectory (blue), and radar trajectories corrected by different methods, demonstrating the superior performance of the ViT variant.

#### 3.3.2. Error Distribution Analysis

**Figure 3:**
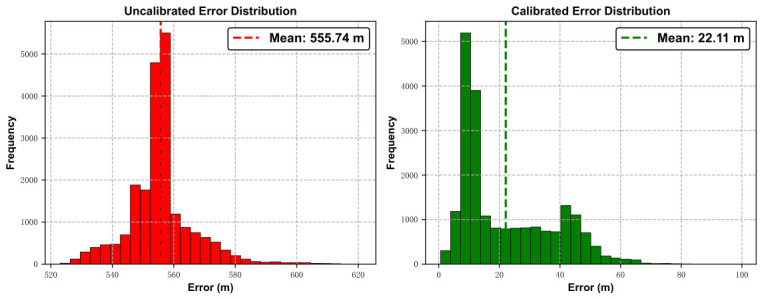
Distribution of residual errors.

Figure 3 presents the distribution of residual errors after error correction. The ViT variant produces a substantially more concentrated error distribution centred around zero, with the trajectory-level mean positioning error reduced from 555.50 m to 6.84 m, demonstrating the effectiveness of the proposed error-correction approach.

**Interpretation of the distribution shape change.** The pre-correction error distribution exhibits an approximately symmetric, bell-shaped profile centred at a non-zero mean (approximately 514.76 m in range, 1.37° in azimuth). This shape arises because the dominant error sources are systematic biases that shift all measurements in a consistent direction: antenna misalignment produces an azimuth-dependent sinusoidal bias; atmospheric refraction introduces a monotonic range-dependent offset; and timing delays in the signal processing chain add a constant range offset. Since these systematic effects apply to all measurements and have relatively low variance compared with their magnitude, the resulting error distribution approximates a shifted normal distribution—the systematic bias determines the mean, and the combined random noise (GPS uncertainty, sea clutter, and vessel motion) contributes to the variance around that mean. The post-correction distribution, by contrast, is sharply concentrated near zero with a narrower spread and reduced symmetry. This transformation is expected and physically meaningful: the model subtracts the learned systematic error component, leaving only the irreducible residual—predominantly AIS GPS uncertainty (5–10 m) and radar thermal noise. The residual does not follow a normal distribution because these noise sources are not identically distributed across all measurements (GPS accuracy varies with satellite geometry, and radar noise is range-dependent), and the model’s correction is imperfect at the tails, where training data are sparser. The near-zero centring and reduced dispersion confirm that the ViT variant successfully extracts the systematic component of the radar error, while the asymmetry and tail structure of the residual reflect the physical characteristics of the remaining noise sources.

### 3.4. Ablation Study

To assess the contribution of each architectural hyperparameter, ablation studies are conducted using a one-factor-at-a-time strategy: each experiment varies a single hyperparameter while holding the others fixed at a base configuration. The base configuration used in these ablation experiments differs from the final optimised model (Section 3.1.3), as the ablations were performed during the hyperparameter search phase to independently identify the best value for each factor. The final model integrates the optimal values identified across all ablation experiments.

#### 3.4.1. Effect of Encoder Layers

**Figure A2:**
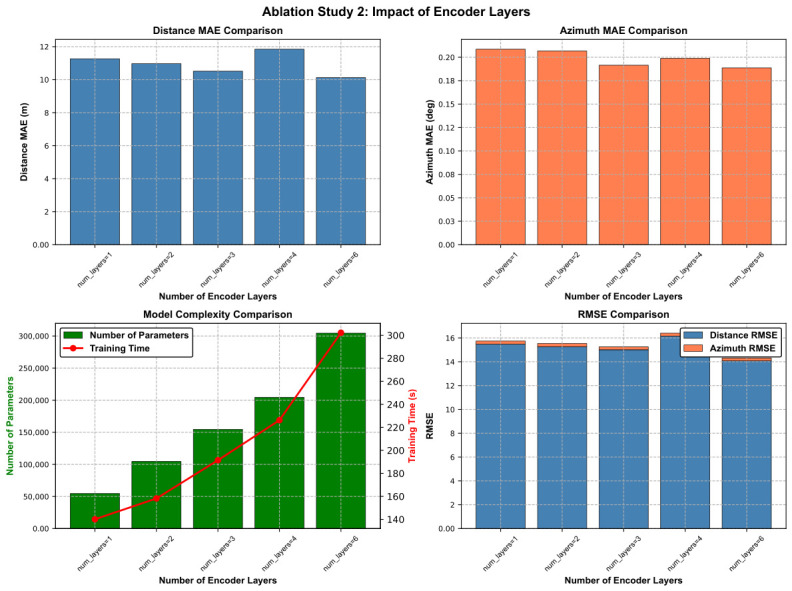
Ablation study: effect of the number of encoder layers.

Figure A2 (Appendix B) illustrates the impact of the number of Transformer encoder layers on error correction performance.

The best performance is achieved with 4 encoder layers, which balances model capacity and computational efficiency. Deeper networks (6 layers) show only marginal improvement, but at the cost of a substantially increased parameter count and longer training time.

#### 3.4.2. Effect of Attention Heads

Table 3 investigates the impact of the number of attention heads on error correction performance. The base configuration uses $d_{\mathrm{model}}=120$, $d_{\mathrm{ff}}=480$, N=3 encoder layers, and patch size = 1.

Based on the experimental results at $d_{\mathrm{model}}=120$, the best performance is achieved with 10 attention heads: it yields the lowest range RMSE (13.86 m) and azimuth MAE (0.19°), while 12 heads achieve an equal range MAE (10.00 m) with slightly higher RMSE and azimuth MAE, but faster training (782.72 s vs. 838.20 s). However, given the marginal differences between 10 and 12 heads, and given that the final model uses a different embedding dimension ($d_{\mathrm{model}}=64$), where 8 heads are employed, the choice of head count is not critical so long as the per-head dimension ($d_{\mathrm{model}}/n_{\mathrm{head}}$) remains in a reasonable range (6–16).

This ablation used $d_{\mathrm{model}}=120$, N=3 layers, a patch size of 1, and 2 raw input features (rather than the full 12) to ensure that $d_{\mathrm{model}}$ was divisible by all tested head counts. The final model (Table 1) performs notably better (range RMSE 11.18 m vs. 13.66 m) because it jointly optimises all hyperparameters: $d_{\mathrm{model}}=64$, 4 encoder layers, patch size 3, and the full 12-feature representation (Section 2.3.3). Each change contributes independently: the extra encoder layer captures higher-order feature interactions; the larger patch size groups related features to produce richer token representations; the 12-feature augmentation provides trigonometric and polynomial terms that encode the angular geometry of radar errors. Together, these improvements account for the 2.48 m range RMSE gap between the ablation base configuration and the final model.

#### 3.4.3. Effect of Patch Size

Table 4 examines the influence of patch size on error correction performance. The base configuration uses $d_{\mathrm{model}}=64$, $d_{\mathrm{ff}}=256$, N=3 encoder layers, and $n_{\mathrm{head}}=4$.

Based on the experimental results, a patch size of 3 achieves the best performance, with the lowest range MAE (9.69 m), range RMSE (13.66 m), and azimuth MAE (0.16°). Smaller patch sizes (1 and 2) yield comparable range accuracy but at substantially higher training cost, while larger patch sizes (4, 6, 12) trade accuracy for faster training. These results indicate that a patch size of 3 strikes an effective balance between fine-grained feature extraction and computational efficiency for the given input dimensionality.

## 4. Discussion

This section analyses the experimental results from methodological, comparative, and practical perspectives, and discusses the limitations of the present approach alongside directions for future work.

### 4.1. Analysis of ViT Variant Effectiveness

The strong error correction performance of the ViT variant stems from three architectural factors that directly address shortcomings of existing methods. First, the multi-head self-attention mechanism computes pairwise interactions across all input tokens, capturing error dependencies that span different range intervals and azimuth sectors—unlike partitioning methods, which assume locally uniform errors and cannot model cross-region correlations. Second, the stacked encoder layers build hierarchical representations that capture both fine-grained local patterns and coarse-grained global trends without manual feature engineering beyond the initial 12-feature set. Third, the multi-task framework with uncertainty weighting jointly optimises range and azimuth prediction, exploiting the physical correlation between these two error dimensions; the learned weights adaptively balance the two tasks, avoiding the fixed-weight heuristics of conventional approaches.

### 4.2. Comparison with Existing Methods

Traditional partitioning methods (UP, NUP) are limited by their reliance on predefined spatial divisions that cannot capture nonlinearly varying errors. Machine learning methods (SVR, XGBoost) improve upon partitioning through data-driven fitting, but their shallow architectures lack the capacity to model long-range feature interactions. STDNN demonstrates the benefit of depth, yet treats range and azimuth errors independently, forgoing the cross-dimensional correlation that the ViT variant exploits through multi-task learning.

#### 4.2.1. Architectural Contribution vs. Training Budget

To disentangle the effects of model capacity and training recipe from genuine architectural innovation, a capacity-matched Transformer baseline was evaluated: four encoder layers, eight attention heads, $d_{\mathrm{ff}}=256$, and the identical training recipe as the ViT variant (AdamW, cosine annealing, uncertainty weighting). It differed only in three architectural aspects: token-per-feature embedding replaces patch embedding, fixed sinusoidal encoding replaces learnable positional encoding, and mean pooling replaces CLS token aggregation.

The capacity-matched Transformer achieves comparable range accuracy (MAE 7.51 m vs. 7.77 m, RMSE 10.88 m vs. 11.18 m; Table 1), while the ViT variant holds modest advantages in azimuth accuracy (MAE 0.14° vs. 0.15°, P95 0.41° vs. 0.43°) and range tail-error robustness (P95 23.01 m vs. 23.16 m). Three conclusions emerge. First, adequate capacity and modern training recipes are necessary conditions for strong performance: all matched-capacity models substantially outperform the undersized vanilla Transformer. Second, the specific architectural choices—patch embedding, learnable positional encoding, and CLS token aggregation—contribute modest but measurable gains, particularly for azimuth correction and tail-error suppression, consistent with the greater nonlinearity of azimuth error patterns. Third, the 12-feature engineered representation (Section 2.3.3) is arguably the single most consequential design choice: the 98.5% range MAE reduction is achieved by all deep models using these features, indicating that physically motivated feature engineering contributes at least as much as any specific architectural decision.

The progressive improvement across the three model variants—Vanilla Transformer → capacity-matched Transformer → ViT variant—can be understood as a cumulative build-up in which gains from each stage are preserved in the next. The three ViT-specific components are functionally orthogonal: patch embedding shapes the input representation, learnable positional encoding controls token ordering, and CLS token aggregation determines how encoder outputs are synthesised into predictions. Because these components operate at distinct stages of the forward pass and address complementary aspects of the learning problem, their effects are largely additive; this orthogonality justifies the one-factor-at-a-time ablation strategy (Section 3.4) and explains why individually optimised configurations combine without adverse interactions.

#### 4.2.2. Comparison with Alternative Architectures

The MLP-Mixer (range MAE 9.20 m, azimuth MAE 0.13°) outperforms traditional methods but trails all Transformer-based models, confirming that self-attention offers a meaningful advantage over pure MLP architectures for this regression task. The TabTransformer achieves the closest performance to the ViT variant (range MAE 7.82 m, range P95 22.83 m), demonstrating that column-wise self-attention is a viable alternative modelling strategy. Several architectures were considered but found inapplicable: Perceiver-style models [38] target high-dimensional inputs and introduce cross-attention bottlenecks unnecessary for 12-dimensional tabular data; state-space models (e.g., Mamba [39]) assume sequential structure absent in our per-observation features; and hybrid CNN-Transformer and Temporal Transformer architectures are designed for grid-structured or time-series inputs, respectively, which differ fundamentally from the static regression problem addressed here.

### 4.3. Practical Implications

The residual range MAE of 7.77 m falls within the intrinsic GPS accuracy of AIS (5–10 m), indicating that the model extracts nearly all correctable systematic error; the remaining discrepancy is dominated by irreducible AIS position uncertainty. The low P95 values (23.01 m range, 0.41° azimuth) confirm that worst-case errors are well controlled, which is critical for collision avoidance where tail behaviour matters more than average accuracy. Cross-validation yields stable performance (range MAE 7.80±0.12 m across 10 seeds), an important attribute for operational deployment with infrequent retraining.

The model contains approximately 205,000 parameters (0.80 MB, float32), requires roughly 2.0 MFLOPs per inference, and runs entirely on CPU. Measured on a desktop CPU, single-sample latency is 2.0 ms (500 samples/s); at a radar update rate of 10 Hz (100 ms interval), this represents under 2% of the available processing window—negligible overhead that enables in-line correction without specialised hardware, even on the embedded processors typical of coastal radar installations.

### 4.4. On the Plausibility of the 98.5% Error Reduction

The magnitude of the error reduction—from 514.76 m to 7.77 m MAE—invites scrutiny regarding data leakage, overfitting, and metric artefacts.

Data leakage is ruled out by the vessel-level split (Section 2.2), which ensures the model is evaluated exclusively on vessels unseen during training, and by grouping temporally adjacent observations into the same split. Overfitting is countered by three observations: cross-validation yields consistent performance across seeds and folds (range MAE 7.80±0.12 m); the sample-to-parameter ratio of approximately 273:1 (56,000 training samples, 205,000 parameters) is favourable for generalisation; and validation loss curves show no divergence from training loss. Physical plausibility is supported by the residual MAE lying at the limit of AIS GPS accuracy (5–10 m): the model extracts the deterministic, learnable systematic biases (antenna misalignment, timing offsets, atmospheric refraction), leaving irreducible AIS noise that cannot be predicted from radar measurements. The 98.5% reduction therefore reflects the ratio of correctable systematic error to total raw error. Metric robustness is confirmed by consistent improvements across all three metrics (MAE, RMSE, P95), and by the independent trajectory-level positioning error, which falls from 555.50 m to 6.84 m after correction. Finally, the learned error map (Section 3.3.2) exhibits smooth, spatially coherent patterns consistent with known physical error sources, rather than the noisy, vessel-specific patterns that would indicate memorisation.

### 4.5. Limitations and Future Work

Four limitations define the scope of this study. First, the evaluation is confined to a single shore-based radar station; generalisation to different systems, locations, and environmental regimes requires multi-site validation. Second, the feature representation uses only geometric quantities derived from the radar measurement; incorporating environmental parameters (temperature, humidity, and sea state) could improve accuracy under adverse conditions, albeit at the cost of additional sensor infrastructure. Third, the model’s internal decision process remains opaque—attention-map analysis, which was not conducted here, is needed to build trust in safety-critical deployment. Fourth, the framework processes each measurement independently; incorporating temporal context through track-level filtering could further suppress residual noise.

Future work will pursue (i) cross-system validation on independent coastal radar datasets; (ii) environmental feature integration to handle adverse propagation conditions; (iii) attention-map analysis to interpret model decisions; (iv) transfer learning to reduce the data requirement for new radar installations; and (v) operational field trials to evaluate robustness under live constraints.

## 5. Conclusions

This work demonstrated that systematic errors in shore-based radar can be effectively corrected by a compact Vision Transformer variant operating solely on radar measurement geometry. Using 12 physics-motivated features processed through self-attention with patch embedding, learnable positional encoding, and CLS token aggregation, the model reduced range MAE from 514.76 m to 7.77 m (98.5%) and azimuth MAE from 1.37° to 0.14° (89.8%), reaching the limit of AIS GPS accuracy. A capacity-matched control experiment confirmed that the specific architectural choices contribute measurable gains in tail-error robustness beyond what increased capacity and modern training recipes alone provide. The model’s compact footprint (approximately 205,000 parameters, 0.80 MB, and 2.0 ms per inference on a CPU) enables deployment on standard hardware without GPU acceleration, making it a practical candidate for integration into existing maritime surveillance pipelines. Future work will focus on cross-system validation, environmental feature integration, and attention-map analysis to improve generalisation and interpretability.

## Figures and Tables

**Table 1:** Overall error correction performance comparison on the test set.

Method	Range Error (m)			Azimuth Error (°)		
	MAE	RMSE	P95	MAE	RMSE	P95
Uncalibrated	514.76	519.40	556.75	1.37	1.53	2.98
UP	43.41	114.83	396.21	0.65	0.83	1.78
NUP	24.01	55.70	118.83	0.57	0.77	1.75
SVR	30.87	40.12	92.09	0.61	0.78	1.76
XGBoost	26.22	34.19	81.36	0.43	0.60	1.36
STDNN	18.00	21.00	39.95	0.30	0.42	0.92
Deep MLP (Eq. Cap.)	9.41	12.44	23.86	0.15	0.22	0.44
MLP-Mixer	9.20	12.52	24.94	0.13	0.19	0.45
TabTransformer	7.82	11.07	22.83	0.15	0.21	0.42
Transformer (Vanilla)	8.01	12.11	28.21	0.15	0.23	0.53
Transformer (Eq. Cap.)	7.51	10.88	23.16	0.15	0.21	0.43
**ViT Variant**	**7.77**	**11.18**	**23.01**	**0.14**	**0.21**	**0.41**

*Note*: “Eq. Cap.” denotes models with capacity matched to the ViT variant (identical training recipe and parameter budget). The horizontal line separates existing baselines from newly introduced models.

**Table 2:** Statistical significance analysis: ViT variant vs. baseline models across 10 independent runs.

Metric	Model A	Mean A ± Std	Model B	Mean B ± Std	Impr.	*p*-Value	Cohen’s *d*
*ViT Variant vs. Transformer (Vanilla)*							
Range MAE (m)	Transformer (Vanilla)	8.01 ± 0.03	ViT	7.77 ± 0.03	3.0%	<$10^{-4}$ ***	0.72 (medium)
Range RMSE (m)	Transformer (Vanilla)	12.11 ± 0.03	ViT	11.18 ± 0.05	7.7%	<$10^{-4}$ ***	0.89 (large)
Range P95 (m)	Transformer (Vanilla)	28.21 ± 0.06	ViT	23.01 ± 0.14	18.4%	<$10^{-4}$ ***	1.21 (large)
Azimuth MAE (°)	Transformer (Vanilla)	0.15 ± 0.01	ViT	0.14 ± 0.01	6.7%	0.008 **	0.48 (small)
Azimuth RMSE (°)	Transformer (Vanilla)	0.23 ± 0.01	ViT	0.21 ± 0.01	8.7%	<$10^{-4}$ ***	0.68 (medium)
Azimuth P95 (°)	Transformer (Vanilla)	0.53 ± 0.01	ViT	0.41 ± 0.01	22.6%	<$10^{-4}$ ***	1.32 (large)

** p<0.01, *** p<0.001. Impr. = relative improvement of Model B over Model A. Cohen’s *d*: |d|<0.2 negligible, |d|<0.5 small, |d|<0.8 medium, |d|≥0.8 large.

**Table 3:** Ablation study: effect of attention heads.

Attention Heads	Range MAE (m)	Range RMSE (m)	Azimuth MAE (°)	Training Time (s)
1 head	11.39	15.33	0.20	1696.64
2 heads	11.59	15.71	0.20	1431.57
4 heads	10.33	14.31	0.19	1985.32
6 heads	11.47	15.70	0.22	1734.30
8 heads	12.18	16.44	0.20	1120.44
**10 heads**	**10.00**	**13.86**	**0.19**	**838.20**
12 heads	10.00	13.95	0.20	782.72

**Table 4:** Ablation study: effect of patch size.

Patch Size	Range MAE (m)	Range RMSE (m)	Azimuth MAE (°)	Training Time (s)
1	9.85	13.70	0.18	2580.28
2	9.88	13.88	0.18	1619.73
**3**	**9.69**	**13.66**	**0.16**	**1578.15**
4	9.93	13.72	0.18	1251.28
6	10.03	13.94	0.18	673.59
12	9.82	13.72	0.17	527.27

## Data Availability

The Maritime Target Detection and Tracking (MTDSP) dataset analysed in this study is publicly available at https://doi.org/10.12000/JR25001.

## Abbreviations

The following abbreviations are used in this manuscript:

AIS	Automatic Identification System
ViT	Vision Transformer
MTDSP	Maritime Target Detection and Tracking
MAE	Mean Absolute Error
RMSE	Root Mean Square Error
IR	Improvement Ratio
UP	Uniform Partitioning
NUP	Non-uniform Partitioning
SVR	Support Vector Regression
STDNN	Single-Task Deep Neural Network
DNN	Deep Neural Network
MSA	Multi-Head Self-Attention
FFN	Feed-Forward Network
LN	Layer Normalisation
SNR	Signal-to-Noise Ratio
CLS	Classification Token

## Appendix A. Trajectory Correction Visualisation

Figure A1 presents the trajectory correction results obtained using different methods. Red lines represent the original radar trajectories, blue lines represent the AIS ground-truth trajectories, and green lines represent the corrected radar trajectories. Figure A1c,d show results for the newly introduced baselines.

## Appendix B. Ablation Study: Effect of Encoder Layers

Figure A2 shows the effect of the number of Transformer encoder layers on error correction performance.
